# Supplementary figures and images for: Autophagy is important to the acidogenic metabolism of Aspergillus niger
Source: PLoS One. 2019 Oct 11;14(10):e0223895. doi: 10.1371/journal.pone.0223895 (PMC6788731; doi:10.1371/journal.pone.0223895)

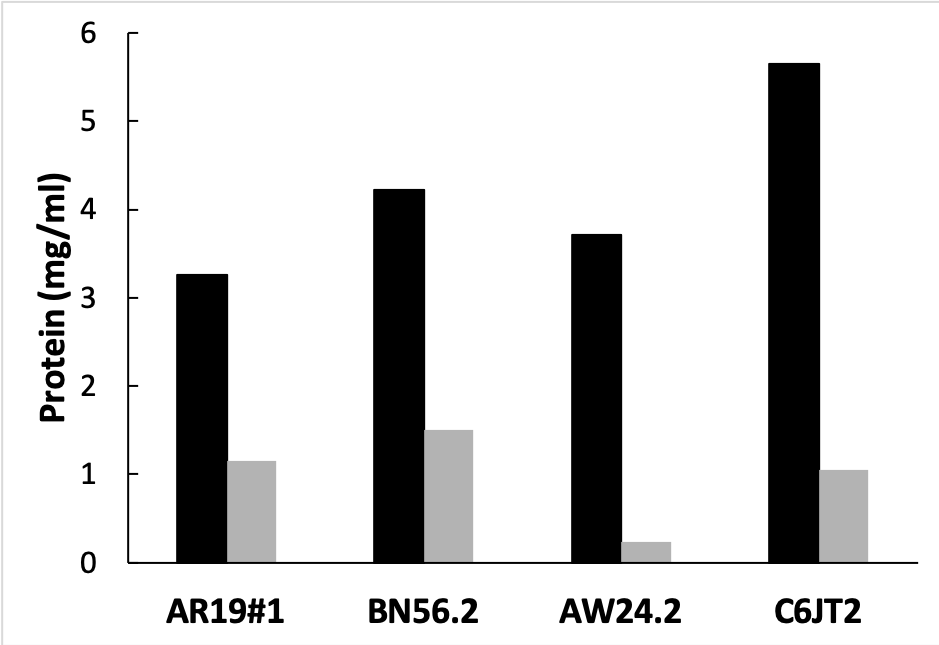

Supplement: S1 Fig — The total protein from the mycelial extracts (2 g wet weight; collected 24 h after transfer to either MM or MM-N) of A. niger strains AR19#1 (wild type), BN56.2 (Δatg1), AW24.2 (atg1 complemented) and C6JT2 is shown (average of two separate experiments). (TIF) [file pone.0223895.s001.tif]

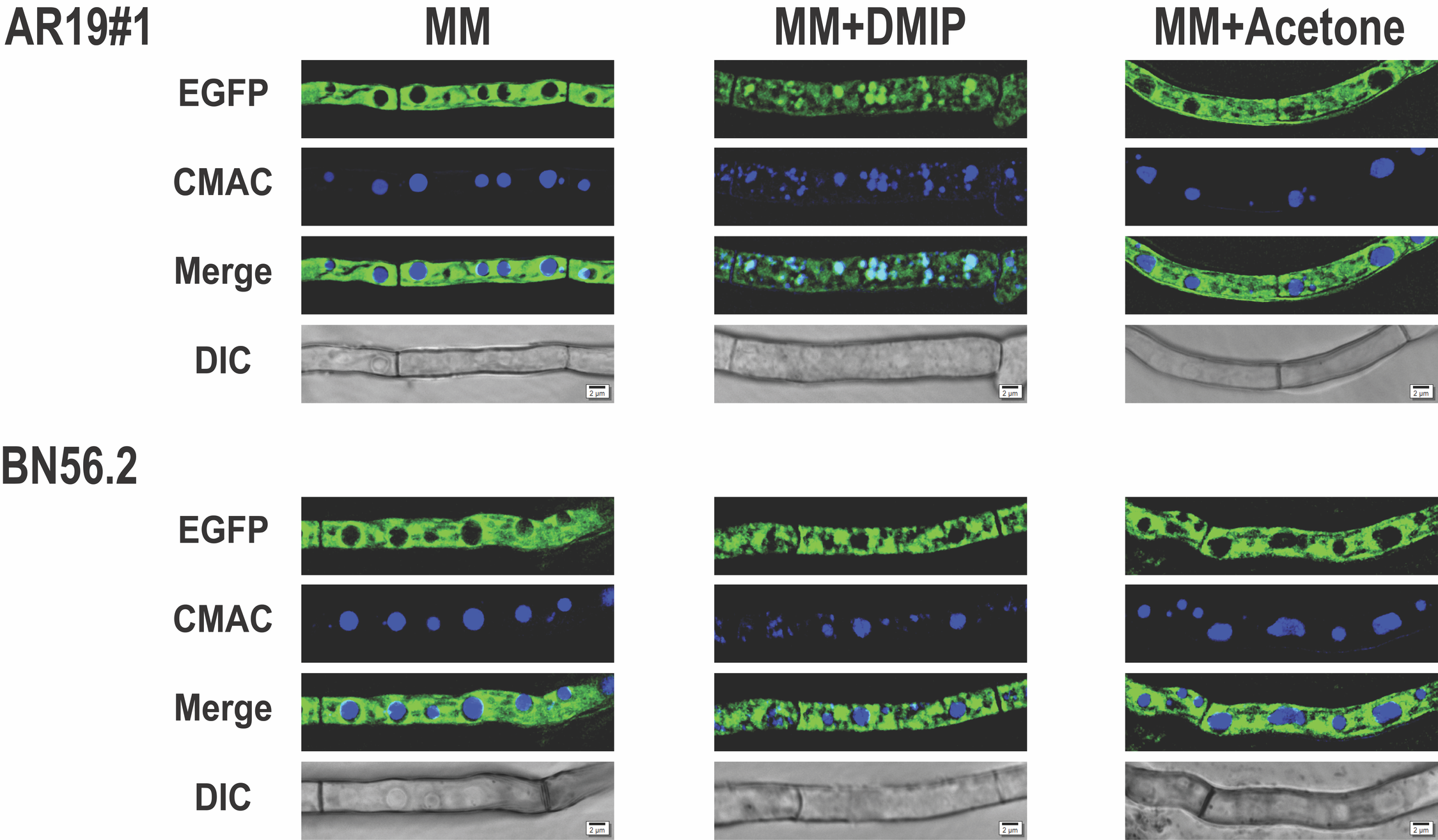

Supplement: S2 Fig — The mycelia of A. niger strains AR19#1 (wild type) and BN56.2 (Δatg1) expressing EGFP in the cytoplasm were grown on minimal medium (MM) till 20 h and then transferred to MM+ DMIP, MM+acetone (solvent control) and MM alone. The EGFP fluorescence of mycelia recorded after 6 h of DMIP treatment is shown. A total of 50 micrograph of each cell type were imaged (scale bar = 2 μm). (TIF) [file pone.0223895.s002.tif]

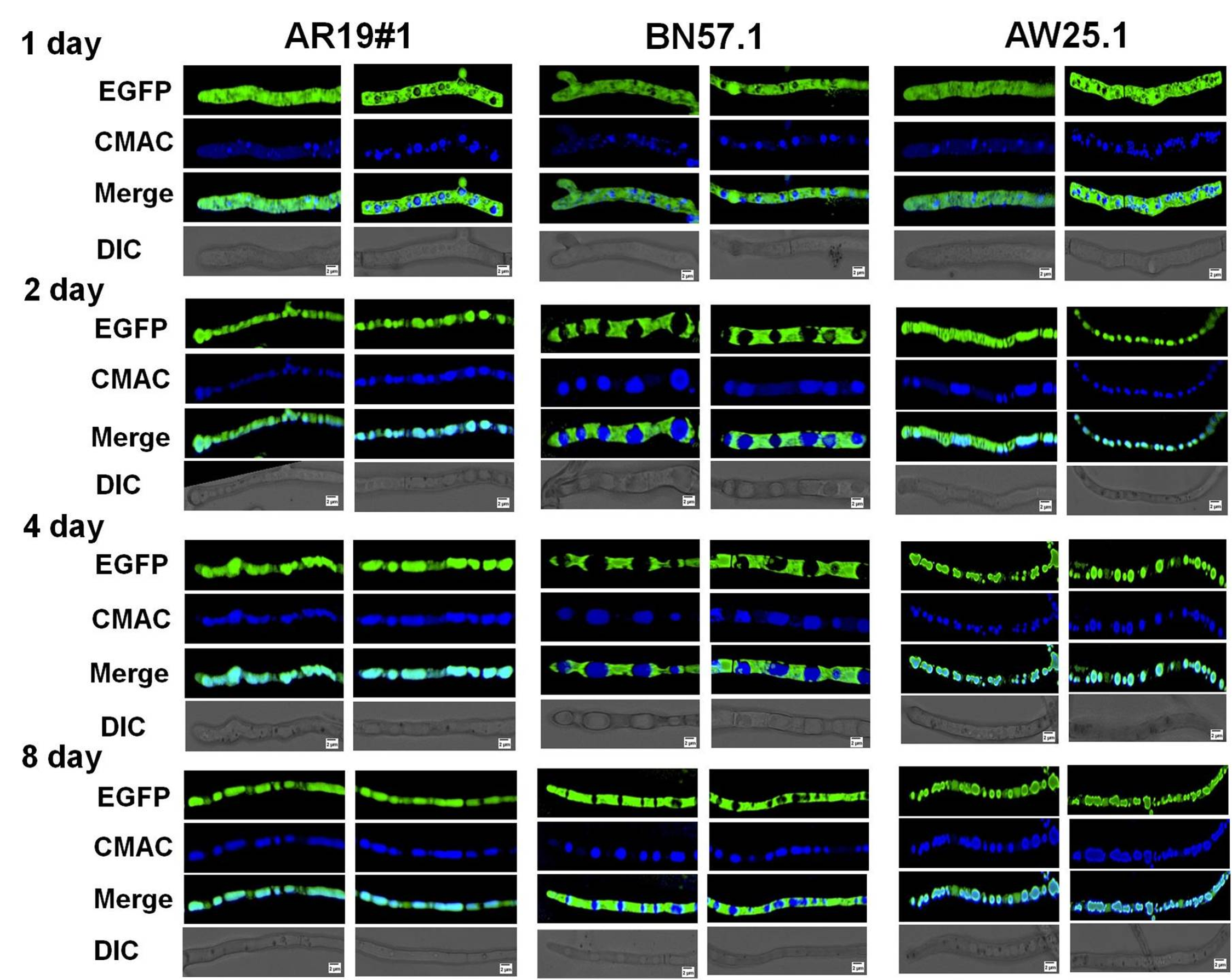

Supplement: S3 Fig — The morphology and EGFP fluorescence of A. niger strains, AR19#1 (wild type), BN57.1 (Δatg8) and AW25.1 (atg8 complemented) was recorded on fermentation medium (AM) for 8 days. In each panel, the left frame shows the image of a tip cell and the right frame is of an intermediate cell. The mycelia were stained with CMAC when vacuoles appear blue; the vacuoles containing EGFP appear cyan on merge (scale bar = 2 μm). (TIF) [file pone.0223895.s003.tif]

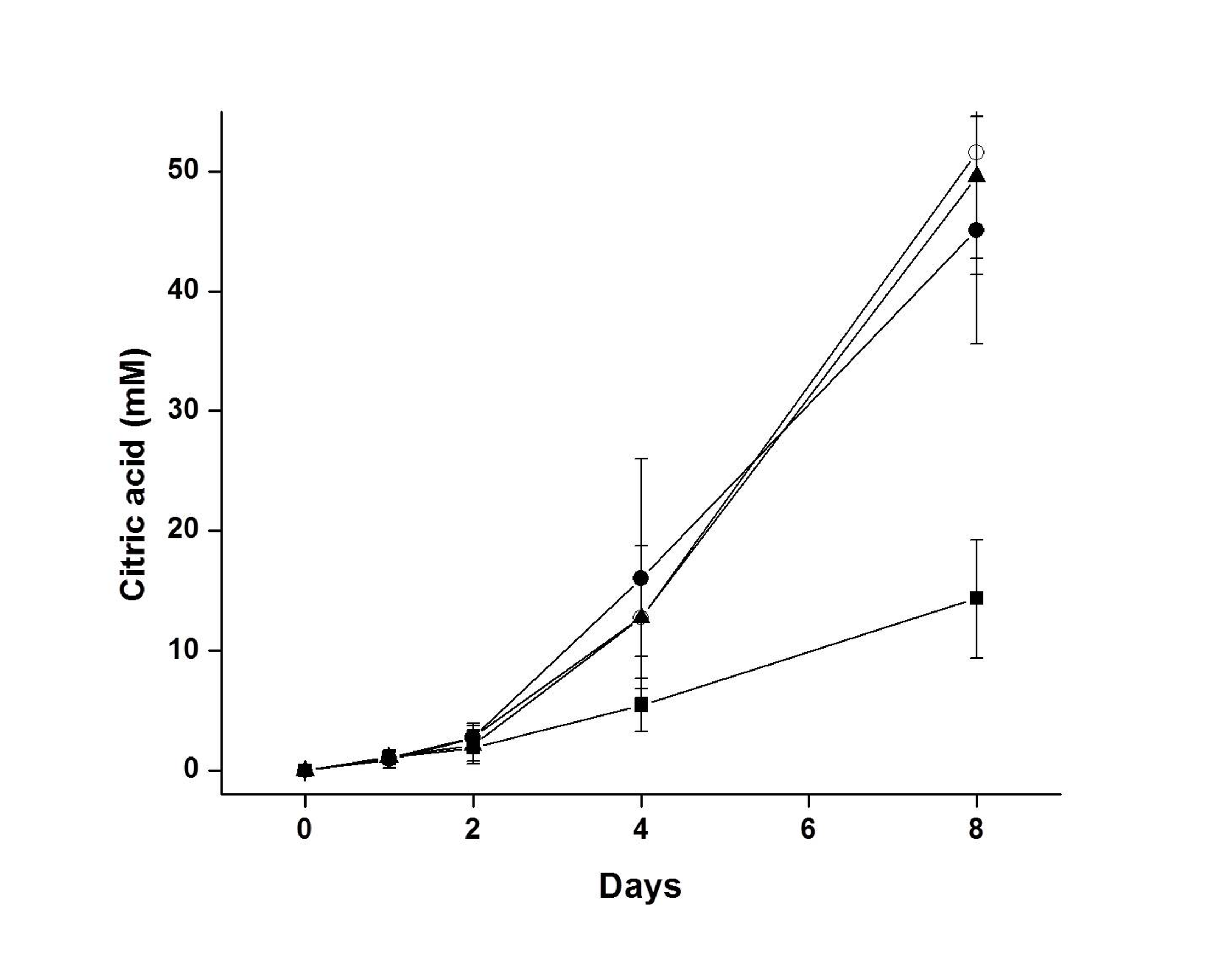

Supplement: S4 Fig — The A. niger strains C6JT2 (○), AR19#1 (●), BN57.1 (■) and AW25.1 (▲) were grown on fermentation medium (AM) and the citric acid measured in the spent medium is shown. (TIF) [file pone.0223895.s004.tif]
