# Supplementary material for: Autophagy is important to the acidogenic metabolism of Aspergillus niger
Source: PLoS One. 2019 Oct 11;14(10):e0223895. doi: 10.1371/journal.pone.0223895 (PMC6788731; doi:10.1371/journal.pone.0223895)
Supplement: S1 Table — (DOC) [file pone.0223895.s005.doc]

**Table S1: Primers used in this work**

| **Primer** | **Description** | **Sequence (**5'→3'**)** |
| --- | --- | --- |
| diagnRP | forward primer specific to 3′ downstream region of *A. niger atg1* | gtcaaggtacgagcttagaatgcagttgc |
| diagnatg1FP | reverse primer specific to *A. niger atg1* | caaccgattgaacgaggtgctggagaagg |
| HYnested | forward primer specific to *hygR* gene | cgatgtaggagggcgtggatatgtc |
| YGnested | reverse primer specific to *hygR* | cgatttgtgtacgcccgacagtcc |
| Atg8FwdP | forward primer specific to *A. niger atg8* | gagcacaaggatgaagatgg |
| ATG8cDNARP | reverse primer specific to 3′ downstream region of *A. niger atg8* | gccaagagagttcacacg |
